# Is ADHD Medication Monitoring Being Completed in CAMHS?

**DOI:** 10.1192/bjo.2024.587

**Published:** 2024-08-01

**Authors:** Benjamin Johnston, Helen McFerran

**Affiliations:** Southern H&SC Trust, Craigavon, United Kingdom

## Abstract

**Aims:**

- To determine the demographics of the patients prescribed medications for ADHD under the CAMHS teams within Southern Trust (NI).
- To assess whether the physical health monitoring guidelines (as outlined by NICE – nice.org.uk/guidance/ng87) have been followed.
- If monitoring is not up to date, to determine why not.

**Methods:**

We reviewed records from clinical notes and NIECR (Northern Ireland Electronic Care Record) to collect demographic details.

Following NICE guidelines, we used the clinical notes to determine which patients had physical health monitoring up to date, including heart rate (HR), blood pressure (BP), weight and height.

For any patient with monitoring not up to date, we reviewed the notes or contacted the practitioners to determine why this was the case.

**Results:**

96 patients were found to be prescribed ADHD medications. Full demographic details were obtained and collated for these patients, including age, sex, diagnosis, co-morbidities, and medication information (e.g. preparation, dose, polypharmacy).

Of the 96 patients, 1 was excluded as their monitoring was carried out by paediatrics.

71 out of the remaining 95 had their monitoring up to date, leaving 24 patients with monitoring not up to date. Of these 24:

- 8 were due to non-attendance
- 4 were due to equipment issues (e.g. faulty/unavailable)
- 3 only had partially completed monitoring (e.g. BP, weight, height but no HR recorded)
- 1 was only reviewed virtually
- 1 had documented completion of monitoring, but no figures documented
- 7 unknown – no reason given.

**Conclusion:**

After 1 patient was excluded, 71/95 patients had monitoring up to date (~75%).

Of the remaining 24, some were due to systemic issues affecting all services, e.g. non-attendance or faulty equipment. However, some were due to issues more easily addressed.

This led to a discussion at a trust-wide patient safety meeting, with the following outcomes:

1. Staff were given a presentation on NICE guidelines for ADHD medication monitoring to ensure knowledge is up to date.
2. The importance of completing all aspects of monitoring and documenting these each time was highlighted.
3. If monitoring could not be completed, the reason must be documented, to avoid further “unknowns”.
4. An agreed plan to schedule monitoring appointments following virtual reviews.
5. Annual re-audit using the same data template, with the aim of improving each year.

